# Evaluation of some quality parameters of cassava starch and soy protein isolate matrices during deep fat frying in soybean oil

**DOI:** 10.1002/fsn3.904

**Published:** 2018-12-19

**Authors:** Adebukola T. Omidiran, Olajide P. Sobukola, Silifat A. Sanni, Lateef O. Sanni, Abdulrasaq A. Adebowale, Adebola O. Shajobi, Peter Kulakow

**Affiliations:** ^1^ Department of Food Science and Technology Federal University of Agriculture Abeokuta Nigeria; ^2^ Department of Nutrition and Dietetics Federal University of Agriculture Abeokuta Nigeria; ^3^ Cassava Breeding Unit International Institute of Tropical Agriculture Ibadan Nigeria

**Keywords:** cassava starch, fried matrices, quality attributes, soy protein isolate

## Abstract

Snack industry is recently focused on the production of snacks with minimal oil content and enhanced quality attributes which prompted the need to study the changes in snack matrices produced from cassava starch processed from three varieties of cassava roots (TMS‐950289, TME‐419, and TMS‐30572) and soy protein isolate blends fried in soybean oil. Effect of frying temperature (170–180°C), frying time (2–4 min), soy protein isolate inclusion level (5%–15%) on proximate composition, color changes, expansion, texture, and sensory attributes of the snacks was investigated. Optimization of process variables was carried out based on a factorial design (2 level by 3 factor) in the Design Expert version 6.0.8, and models were generated showing the relationship between the independent variables and the responses. The desired goal for each constraint (processing conditions) was kept within 170–180°C for 2–4 min, while all responses, except chewiness, expansion, yellowness, and protein, were set at minimum. Evaluation of sensory attributes of the optimized sample was carried out to determine its level of desirability. The optimized frying conditions for matrices produced from starches of TMS‐950289 are 170°C/4 min/5% SPI with desirability value of 0.507; from TME‐419 are 180°C/2 min/5% SPI with desirability value of 0.475 while for those from TMS‐30572 are 170°C/4 min/15% SPI with desirability value of 0.459. At higher SPI level, the protein content was high at 170°C. At 4 min frying time for all the varieties, the moisture content reduces. The most desired optimized fried snack produced from starch of TMS‐30572 (containing 15% SPI) had higher crispness and lower oil content than other optimized fried snacks.

## 1 INTRODUCTION

Snacks are foods manufactured from combination of various ingredients to meet certain nutritional requirements. Kareem et al. (2015) reported that they are often consumed between meals or in place of regular meals while studying some quality attributes of high‐quality cassava–tigernut composite flour and its extruded snacks. Typically, snacks are expected to add to the nutritional intake of individuals and are commonly produced from locally sourced raw materials for easy accessibility and availability. Minimal variations are expected from snacks produced from each frying experiment to the other as reported by Gazmuri and Bouchon (2009). Therefore, there is need to formulate snacks to promote reproducibility, minimize defects, and maintain uniformity in final products. Majorly, wheat is used largely in snacks and pastries due to its uniqueness in providing elasticity property—a function of its gluten, an important constitutent necessary for dough formation. Unfortunately, soy protein does not contain gluten which supports dough formation; nevertheless, its behavior during snacks production needs to be examined to know whether it also exhibits elasticity property that aids dough formation. However, it is necessary to evaluate the matrices formed during the development of an acceptable snack from locally available raw materials. Cassava is grown extensively for its starchy root which is a major source of carbohydrates. Starch is the main ingredient responsible for the expansion and crispness of snacks as reported by Taewee (2011) and is used as thickener in foods that are not subject to rigorous processing conditions. Cassava starch is known to have excellent textural characteristics, high paste viscosity, and clarity. It has a bland or neutral taste which promotes its use in snacks production. Abioye, Ade‐Omowaye, Babarinde, and Adesigbin (2011) stated that soybean (*Glycine max*) is a cheap source of quality protein that is superior to all other plant foods because it has good balance of the essential amino acids and contains reasonable amount of methionine. It can be processed to obtain different products such as soy concentrate, soy flour, soy milk, soy cake, and soybean oil. The oil is extracted from its seeds as a clean oil with little or no perceived odor, and it is low in saturated fat, contains no transfat, and is high in mono and polyunsaturated fats. Soybean oil is people's most favorite choice as edible oil because of its quality, functionality, low cost and also, for being precursors of Omega‐3, Omega‐6, and Vitamin E according to Mounts et al. (1986). Frying process is complex, and it involves many factors, some of which are dependent on the process itself, and others on the food and type of fat used (Saguy & Pinthus, 1995). Deep fat frying adds some unique characteristics to develop snacks with smooth mouth feel, distinct flavor, color, texture, and palatability (Adedeji & Ngadi, 2009). It is one of the most important processes in the preparation of frozen prefried foods, snacks, and fast foods. Desired sensory attributes such as crispiness, crunchy texture, yellowness, and flavor are developed during deep fat frying. According to Franke and Reimerdes(2007), these properties are controlled by the maillard reaction and fat absorption, respectively. Sánchez‐Gimeno, Negueruela, Benito, Vercet, and Oria (2008) reported that the quality of the products cooked by deep fat frying depends not only on the frying conditions such as frying temperature, frying time, food weight, and frying oil volume, but also on oil types and kind of food materials used. Therefore, the objective of this work is to characterize the snacks produced from cassava starch and soy protein isolate blends fried in soybean oil.

## 2 MATERIALS AND METHODS

Three varieties of cassava roots TMS‐30572, TMS‐950289, and TME‐419 were obtained from Cassava breeding unit, International Institute of Tropical Agriculture, Ibadan, Nigeria. The cassava roots were processed to starch at their processing unit. Soy protein isolate, a light yellow powder, was obtained from NutriChem company and soybean oil from Shoprite, Ikeja, Lagos. Knife, bowls, deep fryer, and other materials were obtained from the food processing laboratory, Federal University of Agriculture, Abeokuta. Nigeria.

### 2.1 Preparation of cassava starch

Starch was extracted from freshly harvested roots using the modified procedure of Aseidu (1989). The cassava roots were weighed immediately after harvesting from farm and they were peeled, washed and grated, and the screened starch was allowed to settle and decanted. The final product was packaged in an airtight ziplock bag to prevent moisture and air intake from the atmosphere.

### 2.2 Dough sample preparation

Dough samples were prepared using modified method of Gazmuri and Bouchon (2009). The samples were weighed at the right proportions and mixed in a bowl, and 72 ml of hot water (100°C) was added to 100 g of sample to form a dough. The dough was rolled, kneaded, and cut out to get accurate shape (diameter 10 cm and height 0.25 cm) with a cutter.

### 2.3 Atmospheric frying

Sheeted dough was cut into round shape diameter 10 cm and height 0.25 cm) and fried in the inner frying compartment of a fryer using deep fat frying technique (SAISHO, Model S‐616, China). A lid was used to cover the inner frying compartment while frying was taking place to ensure maximum submersion in the oil. Frying was carried out by dipping the covered frying baskets in the oil that have been maintained at 170 and 180°C for 2 and 4 min. The samples were removed, drained of any surface oil, and then held in a clean stainless flat surface to cool after each frying batch, before packaging in Ziploc packaging films until further use.

### 2.4 Experimental design

A three‐factor experimental setup was used with frying temperature, frying time, and cassava starch and soy protein isolate (CS:SPI) as the independent factors at two levels each as shown in Table 1. The data obtained were analyzed by factorial methodology based on general factorial design (Table 2) to optimize process variables. Eight combinations were generated in random order according to the design.

**Table 1:** Coded values of the independent variables

Variables	Codes	
	−1	1
Frying temperature (^°^C)	170	180
Frying time (min)	2	4
Level of SPI (%)	5	15

**Table 2:** Experimental runs showing different combinations of the independent variables

Runs	Frying temperature (°C)	Frying time (min)	Level of SPI (%)
1	170	4	5
2	180	2	5
3	170	2	15
4	170	4	15
5	170	2	5
6	180	2	15
7	180	4	15
8	180	4	5

### 2.5 Proximate composition

The fried snacks produced were analyzed for moisture, ash, and oil according to AOAC (2000), and the protein content was analyzed using Kjedahl method (AACC, 46‐12.01). The carbohydrate content was obtained by calculating the difference from the sum total.

### 2.6 Expansion analysis

Expansion was determined using the procedures of Maeda and Cereda (2001). Each dough was measured with a vernier caliper before and after frying to determine its diameter. Expansion was then calculated as the difference between the initial diameter before and the final diameter after frying. Values reported are mean of six measurements for each frying operation.

### 2.7 Chewiness and hardness

Chewiness and hardness of the chips were measured using a Universal Testing Machine (Model:M500‐100ATCapacity:100kN, Stable Micro Systems Ltd., Godalming, Surrey UK). Texture parameters (Chewiness [N] and Hardness [N]) were obtained by placing the fried samples on the texture analyzer and using the compressor probe at Probe Diameter (mm): 60 Deformation (%): 2.1 mm for each texture parameter (Da Silva & Moreira, 2008).

### 2.8 Color analysis

The color intensity of the snacks was determined using a chromameter colour measuring system (Konica Minolta CR‐410, Minolta LTD, Japan) as described by Mariscal and Bouchon (2008). The lightness (L*), redness (a*), and yellowness (b*) values were obtained. The analysis was performed in quadriplicate and results presented are average values of each snack using the following expression:

L* is known as the lightness (*L* = 0 (black), *L* = 100 [white]),
a* (−a* = greenness, + a* = redness)
b* (−b* values = blueness, + b* value = yellowness).

### 2.9 Sensory evaluation

Acceptance testing method described by Omidiran et al. (2016) was used to investigate the acceptability of the fried snacks prepared using the optimized frying conditions. Fifty students of Federal University of Agriculture, Abeokuta, Ogun State, Nigeria were engaged as consumer panelists, and they evaluated the sweetness, expansion, crispness, oiliness, color, appearance, and overall acceptance of fried snack. Each sample attribute was rated using a nine‐point Hedonic Scale. The values reported are mean of scores for each attribute, and a radar chart was used to illustrate the results.

### 2.10 Experimental design and data analysis

A 2^3^ general factorial design was used to study the effect and optimize of independent variables namely frying temperature (170 and 180°C), frying time (2 and 4 min), and soy protein isolate (SPI) level (5% and 15%) on some quality attributes of the fried snacks as shown in Tables 1 and 2.

## 3 RESULTS AND DISCUSSIONS

### 3.1 Proximate composition of the fried snacks

**Figure 1:**
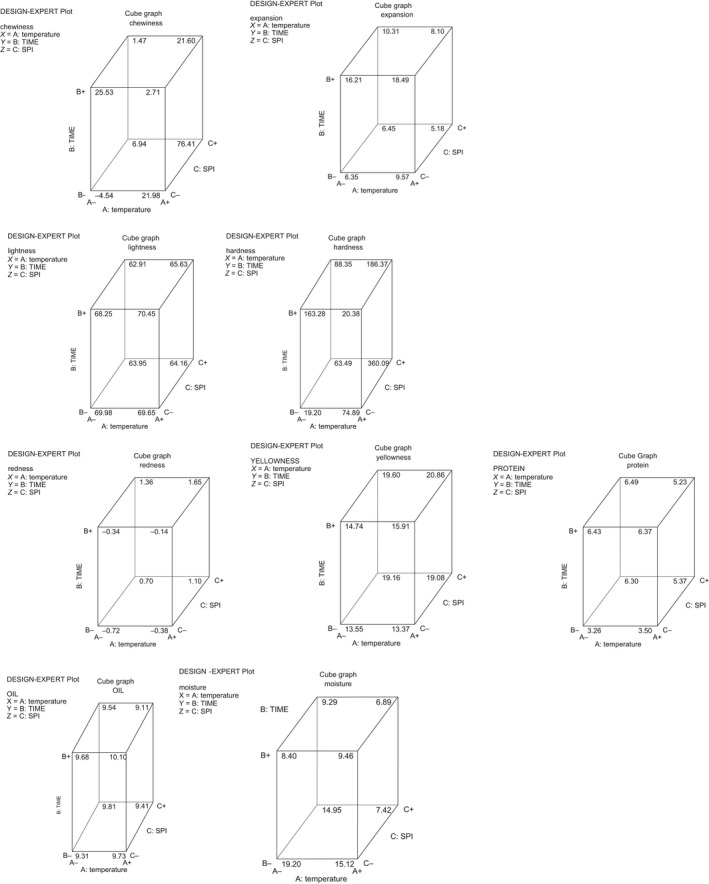
Cube model graphs for responses of snacks from starch of TMS‐950289 and SPI fried in soybean oil

**Figure 2:**
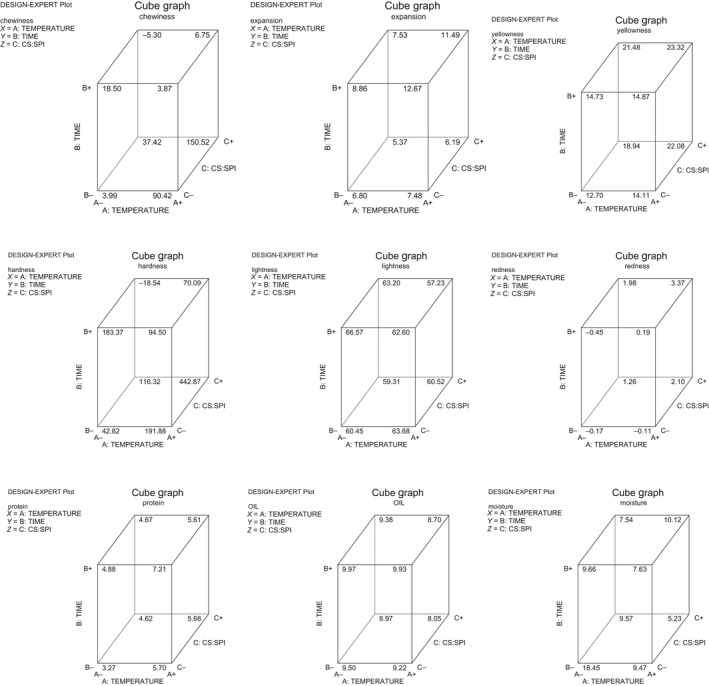
Cube model graphs for responses of snacks from starch of TME 419 and SPI fried in soybean oil

**Figure 3:**
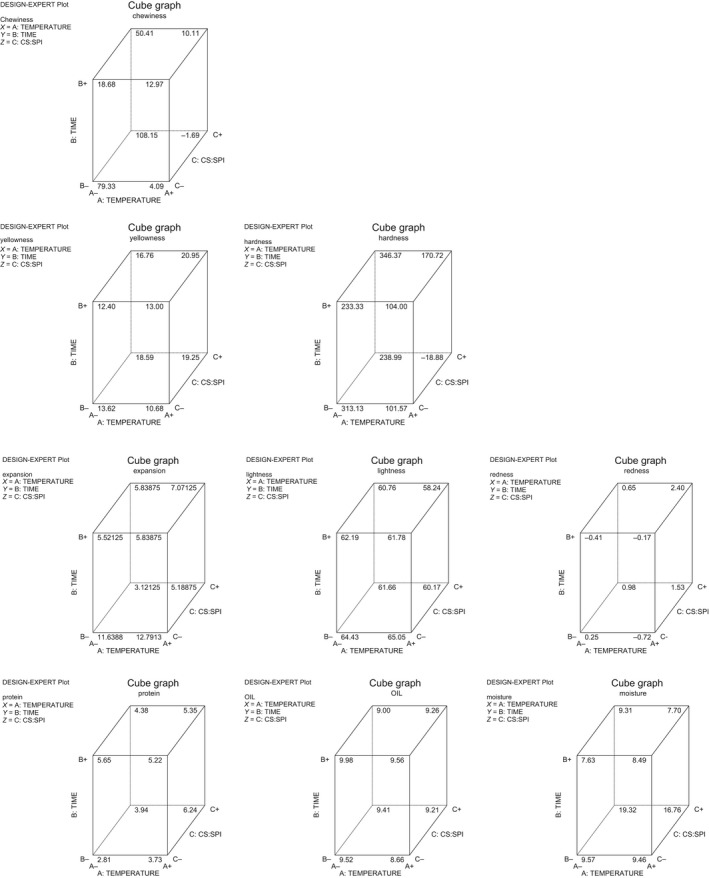
Cube model graphs for responses of snacks from starch of TMS‐30572 and SPI fried in Soybean oil

The protein content ranged from 3.22% to 6.47%, 2.84% to 6.78%, 3.03% to 6.45%; oil content ranged from 9.10% to 10.02, 7.90% to 10.12%, 8.45% to 9.77%, and moisture content ranged from 6.89% to 18.67%, 8.25% to 19.67%, 8.06% to 19.75% for snacks from starches of TMS‐950289, TME‐419, and TMS‐30572, respectively, as shown in Tables 1, 3, and 4, respectively. The coefficient of determination (*R* ^2^) ranged from 0.67 to 0.99, and there were significant (*p *<* *0.05) differences in the protein content as presented in Table 5. High protein content observed in the snacks could be due to the level of soy protein isolate that was supplemented into the starch samples during the snack production. It was observed that the oil content of the snacks reduced with increased frying time. The results may be explained by the formation of a crust, which acts as a barrier to reduce the oil uptake. The crust formation prevents the water molecules bound within the matrices of fried snacks from escaping to the outside and consequently preventing further oil uptake since oil absorption is affected by the porosity of the product. This is supported by the report of Kawas and Moreira (2001) which indicated that though porosity increases during frying but longer frying times resulted in uniform pore size distribution. Moisture content is one of the critical properties used in determining the shelf stability of product. The lower the initial moisture content of a product, the better the storage stability of the product as stated by Akubo (1997), and it was observed in this study that the higher the frying time, the lower the moisture content. So therefore, products fried at 4 min tend to be crunchier. The cube model graphs for the snacks produced from the three varieties are presented in Figures 1, 2, 3 showing the relationship with each quality parameter.

**Table 3:** Responses of snacks from starch of TMS‐950289 and SPI fried in soybean oil

RUN	A	B	C	D	E	F	G	H	I	J	K	L
1	170	4	5	19.76	145.57	16.26	69.45	−0.27	14.67	6.47	9.89	8.93
2	180	2	5	16.20	57.18	9.62	70.85	−0.31	13.3	3.54	9.94	15.65
3	170	2	15	1.16	45.78	6.50	65.15	0.77	19.09	6.34	10.02	15.48
4	170	4	15	7.25	106.06	10.26	61.71	1.29	19.67	6.45	9.33	8.76
5	170	2	5	1.23	36.91	6.30	68.78	−0.79	13.62	3.22	9.10	18.67
6	180	2	15	82.19	377.80	5.13	62.96	1.03	19.15	5.33	9.20	6.89
7	180	4	15	15.83	168.67	8.15	66.83	1.72	20.79	5.27	9.32	7.42
8	180	4	5	8.49	38.09	18.44	69.25	−0.21	15.98	6.33	9.89	8.93

A: temperature (°C); B: time (min); C: SPI (%); D: chewiness (N); E: hardness (N); F: expansion (MM); G: lightness; H: redness; I: yellowness (%); J: protein (%); K: oil (%); L: moisture (%).

**Table 4:** Responses of snacks from starch of TME 419 and SPI fried in soybean oil

RUN	A	B	C	D	E	F	G	H	I	J	K	L
1	180	2	5	83.751	153.723	7.85	64.68	−0.10	14.34	6.13	9.37	8.20
2	170	2	15	30.752	78.165	5.74	60.31	1.27	19.17	5.05	9.12	8.35
3	180	2	15	157.192	481.02	5.82	59.52	2.09	21.58	5.25	7.90	6.45
4	170	2	5	10.662	80.975	6.43	59.45	−0.18	12.47	2.84	9.35	19.67
5	180	4	15	0.082	31.94	11.86	58.23	3.38	23.55	6.04	8.85	8.90
6	180	4	5	10.541	132.655	12.30	61.60	0.18	14.64	6.78	9.78	8.85
7	170	4	5	11.828	145.22	9.23	67.57	−0.44	14.96	5.31	10.12	8.44
8	170	4	15	1.371	19.615	7.16	62.20	1.97	21.25	4.24	9.23	8.76

A: temperature (°C); B: time (min); C: C: SPI (%); D: chewiness (N); E: hardness (N); F: expansion (MM); G: lightness; H: redness; I: yellowness (%); J: protein (%); K: oil (%); L: moisture (%).

**Table 5:** Regression coefficient of the responses as a function of the independent variables of TMS‐950289

Factor	*X*_1_	*X*_2_	*X*_3_	*X*_4_	*X*_5_	*X*_6_	*X*_7_	*X*_8_	*X*_9_
Intercept	19.01	122.01	10.08	66.87	0.40	17.03	5.37	9.59	11.34
A	11.66	38.43	0.25	0.60	0.15	0.27	−0.25	1250E‐003	−1.62
B	−6.18	−7.41	3.19^a^	−0.063	0.23	0.74	0.76^a^	0.021	−2.83
C	7.59	52.57	−2.57^a^	−2.71	0.80	2.64^a^	0.48^a^	−0.12	−1.70
AB	−12.34	−49.64	−0.23	0.63	−0.031	0.34	−0.079	−3.750E−003	1.28
AC	10.74	60.23	−1.12^a^	0.13	0.019	0.024	−0.30	−0.21	−0.86
BC	−8.89	−29.80	−1.50^a^	0.17	0.074	−0.19	−0.75^a^	−0.16	1.28
*R* ^2^	0.945	0.973	0.999	0.850	0.993	0.999	0.999	0.666	0.9843
Std. dev.	49.74	180.20	0.20	3.39	0.21	0.20	0.10	0.58	1.49
*F*‐value	2.89	5.99	1,363.65	0.95	22.09	254.51	193.60	0.33	10.48

A: frying temperature; B: frying time; C: level of SPI; *X*_1_: Chewiness; *X*_4_: lightness; *X*_7_: protein; *X*_2_: hardness; *X*_5_: redness; *X*_8_: oil; *X*_3_: expansion; *X*_6_: yellowness; *X*_9_: moisture.

a Significant at 5% level.

### 3.2 Chewiness, hardness and expansion of fried snacks

The mean values of chewiness ranged from 1.16 to 82.19 N, 0.08 to 157.19 N, 1.23 to 105.26 N while hardness ranged from 36.91 to 377.79 N, 19.62 to 153.72 N, 36.91 to 377.80 N for fried snacks from starches of TMS‐950289, TME‐419, and TMS‐30572, respectively. Crispness is one important quality factor of desirable textural attribute of fried foods because it signifies freshness and high‐quality snacks. It can be observed that increased frying temperature, frying time, and reduced SPI level resulted in an increase in hardness value. Increase in hardness value could be attributed to the lower moisture content of the product. According to Hindra and Baik (2006), higher oil temperature produced faster changes of hardness and accelerated crust formation. Esan, Sobukola, Sanni, Bakare, and Munoz (2015) while studying the quality attributes of vacuum fried yellow‐fleshed sweetpotato chips reported that textural changes during frying are influenced by starch content, size of starch granules, cell wall polysaccharides, nonstarch polysaccharides, and pectic substances. At the beginning of frying, the texture of the fried snacks became softer due to the combination effects of the loss cell integrity, free diffusion of cellular content throughout the tissue, reduction of cell adhesion, and starch gelatinization. As the fried snack becomes crispier, the breaking force reduces and this could be made possible by increasing the frying time and temperature. The texture (hardness) values reported as breaking force reflected the crispiness of the snacks which is the most desired attributes of crisps and chips that denotes freshness and high quality. At higher frying temperature and time, texture also reduced with increased level of SPI. There were no significant effects on the chewiness and hardness of the fried snacks.

**Figure 4:**
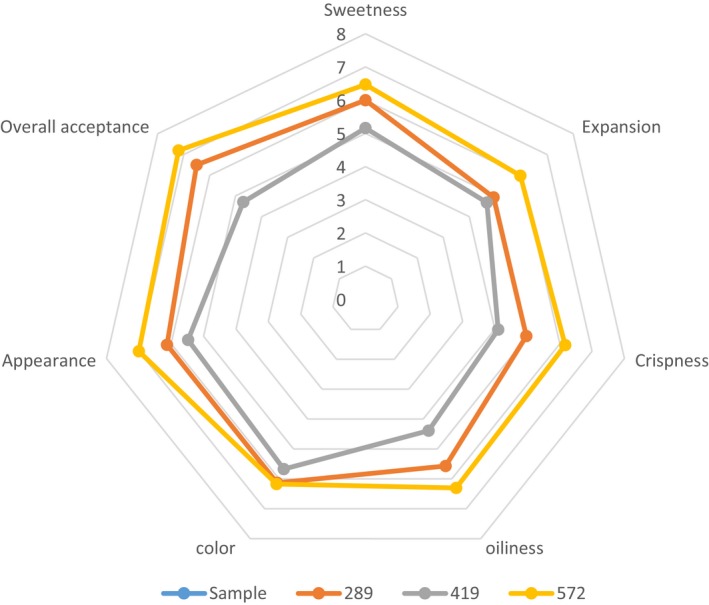
Radar chart showing the level of acceptability of the optimized fried snack in soybean oil based on their attributes using starches from different varieties of cassava

The mean value of expansion ranged from 5.13 to 18.44 mm for fried snacks from starches of TMS‐950289. The model for expansion had the coefficient of determination (*R* ^2^) value of 0.99 and *F*‐value of 1,363.65. Frying time significantly (*p* < 0.05) affected the expansion positively, and SPI significantly (*p* < 0.05) affected the expansion negatively. Furthermore, the interaction between frying temperature and SPI, and frying time and SPI had a significant effect (*p* < 0.05) on the expansion of TMS‐950289 negatively. The mean value of the expansion ranged from 5.74 to 11.86 mm for fried snacks from starches of TME‐419. The model for expansion had the coefficient of determination (*R* ^2^) value of 0.98 and *F*‐value of 6.97 as presented in Table 6. For fried snacks from starches of TMS‐30572, the mean value of the expansion ranged from 3.28 to 12.95 mm, and the model for expansion had the coefficient of determination (*R* ^2^) value of 0.99 and *F*‐value of 64.60. However, the interaction of frying time and SPI had a significant effect (*p* < 0.05) on the expansion of TMS‐30572 as presented in Table 7. Expansion decreased because soy protein isolate and cassava starch do not contain gluten that will support maximum expansion. Increase in SPI substitution level reduced the expansion of the fried snacks. Gazmuri and Bouchon (2009) and Sobukola, Babajide, and Ogunsade (2012) while working on fabricated matrices from wheat starch and gluten reported that products containing high amount of gluten and water tend to expand during frying with the gluten content of the matrix developing an elastic structure that traps water vapor producing an expanded product. This is also supported by the report of Omidiran et al. (2016) on the expansion of fried snacks from blends of wheat flour and brewers’ spent cassava flour.

**Table 6:** Regression coefficient of the responses as a function of the independent variables of TME‐419

Factor	*X*_1_	*X*_2_	*X*_3_	*X*_4_	*X*_5_	*X*_6_	*X*_7_	*X*_8_	*X*_9_
Intercept	38.27	140.41	8.30	61.70	1.02	17.78	5.21	9.22	9.71
A	24.62	59.42	1.16	−0.69	80.37	0.82	0.85	−0.24	−1.60
B	−32.32	58.06	1.84	0.70	0.25^a^	0.82	0.39	0.28	−0.97
C	9.08	12.27	−0.65	−1.63	1.16^a^	3.68^a^	−0.062	−0.44	−1.59
AB	−25.26	−59.48	0.78	−1.80	0.14	−0.32	−0.028	0.060	1.73
AC	6.67	44.37	0.036	−0.50	0.19^a^	0.43	−0.35	−0.16	1.16
BC	−14.31	−68.85	0.026	−0.55	0.25^a^	0.12	−0.39	−0.015	1.69
*R* ^2^	0.983	0.922	0.977	0.881	1.000	0.996	0.862	0.941	0.898
Std. dev.	49.74	180.20	0.47	6.87	1.33	0.45	1.21	0.42	3.46
*F*‐value	9.82	1.98	6.97	1.23	7,045.75	49.35	1.04	2.66	1.47

A: temperature; B: time; C: SPI; *X*_1_: chewiness; *X*_4_: lightness; *X*_7_: protein; *X*_2_: hardness; *X*_5_: redness; *X*_8_: oil; *X*_3_: expansion; *X*_6_: yellowness; *X*_9_: moisture.

a Significant at 5% level. A: frying temperature; B: frying time; C: level of SPI.

**Table 7:** Regression coefficient of the responses as a function of the independent variables of TMS‐30572

Factor	*X*_1_	*X*_2_	*X*_3_	*X*_4_	*X*_5_	*X*_6_	*X*_7_	*X*_8_	*X*_9_
Intercept	35.26	186.15	7.13	61.78	0.56	15.66	4.66	9.33	11.03
A	−28.89	−96.80	0.60	−0.48	0.20	0.31	0.47	−0.15	−0.43
B	−12.21	27.45	−1.06	−1.04	0.054	0.12	0.49	0.12	−2.75
C	6.49	−1.85	−1.82	−1.58	0.83	3.23	0.31	−0.11	2.24
AB	17.38	20.56	−0.21	−0.26	0.30	0.88	−0.34	0.11	0.24
AC	−8.65	−11.58	0.23	−0.53	0.38	0.90	0.35	0.17	−0.61
BC	0.73	46.79	2.21^a^	0.34	0.081	−0.15	−0.60	−0.21	−2.02
*R* ^2^	0.994	0.755	0.997	0.990	0.986	0.993	0.961	0.761	0.990
Std. dev.	10.16	93.19	0.21	4.18	0.14	0.58	0.38	0.97	0.87
*F*‐value	28.71	0.51	64.60	16.89	11.40	22.72	4.10	0.53	15.76

A: temperature; B: time; C: SPI; *X*_1_: chewiness; *X*_4_: lightness; *X*_7_: protein; *X*_2_: hardness; *X*_5_: redness; *X*_8_: oil; *X*_3_: expansion; *X*_6_: yellowness; *X*_9_: moisture.

a Significant at 5% level. A: frying temperature; B, frying time; C: level of SPI.

### 3.3 Color parameters of fried snacks

Tables 3, 4, and 8, respectively, show the mean value of redness value for fried snacks from starches of TMS‐950289, TME‐419, and TMS‐30572, and it ranged from 61.71% to 70.85%, −0.44% to 3.38%, −0.60% to 2.52%. The mean values for lightness ranged from −0.79% to 1.72%, 58.23% to 67.57%, 58.03% to 64.84%, and 13.30% to 20.79%, 12.47% to 23.55%, 12.70% to 21.25% for yellowness of fried snacks from starches of TMS‐950289, TME‐419, and TMS‐30572. Changes observed in the color of fried snacks were as a result of maillard reactions which depend on the content of reducing sugars and amino acids at the surface, the temperature, and the frying time as reported by (Marquez & Anon*,* 2000). As the frying temperature increased, the lightness parameter of the fried product decreased, whereas the redness and yellowness parameters increased for the same frying time. This is similar to the reports of Krokida & Oreopoulou, 2000 and Moyano, Vioseco, & Gonzalez, 2002. Lightness value of fried snacks decreased with increase in frying temperature, frying time, and level of SPI, while redness and yellowness values increased.

**Table 8:** Responses of snacks from starch of TMS‐30572 and SPI fried in soybean oil

RUN	A	B	C	D	E	F	G	H	I	J	K	L
1	170	2	15	105.299	174.33	3.28	61.45	1.1	18.89	3.72	9.20	19.75
2	180	4	15	7.251	106.055	7.23	58.03	2.52	21.25	5.13	9.05	8.13
3	180	2	5	1.232	36.91	12.95	64.84	−0.60	10.98	3.51	8.45	9.89
4	170	4	5	15.825	168.665	5.68	61.98	−0.29	12.70	5.43	9.77	8.06
5	170	2	5	82.188	377.795	11.48	64.63	0.13	13.32	3.03	9.73	9.14
6	180	4	5	15.825	168.665	5.68	61.98	−0.29	12.70	5.43	9.77	8.06
7	180	2	15	1.162	45.78	5.03	60.37	1.41	18.95	6.45	9.42	16.33
8	170	4	15	53.267	411.035	5.68	60.96	0.53	16.46	4.60	9.21	8.88

A: temperature (°C); B: time (min); C: C: SPI (%); D: chewiness (N); E: hardness (N); F: expansion (MM); G: lightness; H: redness; I: yellowness (%); J: protein (%); K: oil (%); L: moisture (%).

### 3.4 Optimization of process variables

Chewiness, expansion, yellowness, and protein content were maximized for each varieties, while hardness, lightness, redness, oil content, and moisture content were minimized for fried snacks from each varieties. Frying temperature of 170°C, frying time of 4 min, and level of SPI of 5% with a desirability of 0.507 were selected for TMS‐950289. Frying temperature of 180°C, frying time of 2 min, and level of SPI of 5% with a desirability of 0.475 were selected for TMEB‐419. Frying temperature of 170°C, frying time of 4 min, and level of SPI of 15% with a desirability of 0.459 were selected for TMS‐30572, and an optimized sample was prepared under these conditions.

### 3.5 Sensory evaluation

The result of sensory evaluation is shown in Figure 4. Sensory evaluation showed that the snacks produced were not significantly different at 5% level. All the samples were found to be acceptable by the panelists because their scores were above average. The sample TMS‐ 30572 (170°C, 4 min, 15%) was rated highest in terms of sweetness (6.47), expansion (5.97), crispness (6.17), oiliness (6.30), color (6.17), appearance (7.00), and overall acceptability (7.20).

## 4 CONCLUSION

It was observed in this study that expansion was significantly affected by the inclusion of soy protin isolate. As the level of SPI increased, it reduced expansion but there was no significant effect on hardness and chewiness. There was decrease in oil and moisture contents of the fried snacks. The frying temperature of 170°C, frying time of 4 min, and level of SPI of 15% were selected to give values for the various responses with desirability value of 0.459 for TMS‐30572, while the frying temperature of 180°C, frying time of 2 min, and level of SPI of 5% with desirability value of 0.475 were selected for TMEB‐419, and also the frying temperature of 170°C, frying time of 4 min, and level of SPI of 5% with desirability value of 0.507 were selected for TMS‐950289. The most desired optimized fried snack (containing 15% SPI) had high crispness, and lower oil content than other optimized fried snacks.

## CONFLICT OF INTEREST

The authors declare that they do not have any conflict of interest.

## ETHICAL REVIEW

This study does not involve any human or animal testing.
